# Knowledge, safety, and impact of alcohol consumption in young adults with type 1 diabetes mellitus: a qualitative study

**DOI:** 10.1186/s12902-023-01471-7

**Published:** 2023-10-20

**Authors:** Oscar T Sergel-Stringer, Hesham S Al-Sallami, Sara E Styles, Alisa Boucsein, Martin I de Bock, Benjamin J Wheeler

**Affiliations:** 1 Department of Women’s and Children’s Health, Dunedin School of Medicine, University of Otago, Dunedin, 9016 New Zealand; 2 School of Pharmacy, University of Otago, Dunedin, 9016 New Zealand; 3 Department of Human Nutrition, Division of Sciences, University of Otago, Dunedin, 9016 New Zealand; 4 Department of Paediatrics, University of Otago, Christchurch, 8011 New Zealand

**Keywords:** Alcohol, Type 1 diabetes, Self-management, Education

## Abstract

**Purpose:**

To explore the lived experiences of alcohol consumption among young adults with type 1 diabetes.

**Methods:**

Fourteen semi-structured interviews were conducted amongst young adults aged between 18 and 25 years, inclusive, with type 1 diabetes and experience consuming alcohol. Interviews were transcribed verbatim and analysed to identify common themes regarding their experiences.

**Results:**

The interviews confirmed that young adults with type 1 diabetes engage in social, and occasionally excessive, drinking behaviour. Furthermore, the interviews revealed four key themes: (i) Several sources contribute to a widely inconsistent understanding of the impact and management of alcohol consumption; (ii) Perceived inconvenience of maintaining healthy glycaemic control whilst drinking socially; (iii) Engagement in proactive strategies for harm reduction occurred when convenient; and (iv) Impact of modern diabetes technology in overcoming previous burdens and promoting glycaemic safety.

**Conclusion:**

Young adults with type 1 diabetes continue to need anticipatory education surrounding safe alcohol consumption and behaviours, as well as ongoing support and encouragement to ensure engagement with traditional self-management tasks. Significant alcohol-diabetes related safety issues, particularly hypoglycaemia do occur, and were captured within this small sample and study. Diabetes technology has an important complementary role along with education and tailored support strategies to support health and safe glucose control during alcohol consumption.

**Supplementary Information:**

The online version contains supplementary material available at 10.1186/s12902-023-01471-7.

## Novelty Statement (bulleted)

Young adults with type 1 diabetes appear to consume alcohol at comparable rates to their counterparts without diabetes, but may experience a higher rate of harm.

Alcohol can result in significant harm (e.g., hypoglycaemia) in young adults with type 1 diabetes.

Current anticipatory education is inconsistent resulting in an increased exposure of some individuals to avoidable harms.

Safety-promoting behaviours can be implemented so long as they are minimally restrictive socially. Furthermore, advanced diabetes technologies potentially may offer a significant harm-reduction benefit to these young adults.

**Supplementary Information:**

The online version contains supplementary material available at 10.1186/s12902-023-01471-7.

## Introduction

Type 1 diabetes is one of the most common chronic medical conditions diagnosed in adolescents and young adults [1]. For individuals with established type 1 diabetes, young adulthood coincides with a period of commonly suboptimal glucose levels [2–4]. Further compounding the risk of diabetes complications, young adulthood is also characterised by increased risk-taking and experimental behaviours which originate during adolescence and peak during adulthood such as substance use, dangerous driving and unprotected sex [5]. The reasons for this are varied, and include navigation of societal role and independence [6], and a neurodevelopmental phase noted for high dopaminergic reward sensitivity, which may drive thrill-seeking and risk-taking [7].

Alcohol consumption also begins to increase during this life phase and is closely linked to thrill-seeking and risk-taking behaviour. Alcohol consumption, even in individuals without type 1 diabetes, predisposes young adults to harms such as increased chance of being either a victim or perpetrator of violence, as well as an increase in the rates of hospitalised injuries, motor vehicle accidents, and sexual risk-taking [8]. International data confirms hazardous drinking behaviours are most common in young adults, especially in higher-income countries [9].

A review of alcohol and recreational drug use in young adults with type 1 diabetes found that the rates of alcohol consumption are generally comparable to other young adults; however, the harms of consumption were significantly greater as observed by inordinate acute hospital admissions induced by alcohol [10]. This is because alcoholic drinks contain varying amounts of carbohydrates and ethanol, which may alter glucose metabolism and increase the risk of hypoglycaemia through multiple mechanisms [11]. For example, a study found that alcohol consumption in people with type 1 diabetes resulted in greater impaired hypoglycaemia awareness, which can predispose those who consume alcohol to delayed intervention and an increased risk of fatal outcomes [12].

Currently, recommendations from the American Diabetes Association include a maximum alcohol suggestion of moderate consumption levels defined as one standard drink (14 g of ethanol) per day for women, and 2 standard drinks per day for men, along with ensuring carbohydrate and calorie content are known [13]. This level of consumption is the same as general public health messaging despite the additional safety risk that alcohol consumption by people with type 1 diabetes confers [14]. Further suggestions from the New Zealand Ministry of Health recommends no more than 4 standard drinks on a single occasion for women, and no more than 5 for men [15]. Despite these guidelines, data from the New Zealand Health Survey has found that 31% of those aged between 18 and 24 are classified as hazardous drinkers [16]. It is currently not known what knowledge young adults with type 1 diabetes have of these guidelines or the impact of alcohol on their diabetes control. Therefore, in this qualitative study we aimed to explore the lived experiences of young adults with type 1 diabetes regarding alcohol and their diabetes management.

## Methods

### Participants

Young adults with type 1 diabetes who had previously been involved in research through the University of Otago, Dunedin, New Zealand, and consented to being contacted for future research were invited to participate in this qualitative study. Inclusion criteria were an age of 18–25 years, confirmed diagnosis of type 1 diabetes, and current or past consumption of alcohol. Those interested in participating were provided with study information and completed standard informed consent.

### Data collection

Between November 2021 – July 2022, semi-structured qualitative interviews were conducted over video conferencing software (Zoom^™^) or in person by OSS; a non-clinical investigator with training in conducting qualitative interviews. Topics included in-depth discussion surrounding participants’ understanding and management of type 1 diabetes with alcohol consumption. Additional topics included their understanding of the effect of alcohol on their glycaemic levels, the frequency of social occasions when alcohol is consumed, the amount and type of alcohol consumed typically, the changes in glycaemic management whilst drinking, the activities they engage in whilst drinking, and details about any past alcohol-diabetes related adverse events experienced. A brief interview guide (supplementary material 1), developed before beginning the study, was used to guide the general discussion of the interview. All interviews were recorded and transcribed verbatim, checked for accuracy, and de-identified. The interviews varied in length between 20 and 40 min and no repeat interviews were conducted. Study participants were given the opportunity to review their interview transcript prior to analysis; two participants opted to review, and no changes were made. Additional demographic information, including clinical values such as HbA_1_C, was self-reported.

### Data Analysis

The general inductive analysis(17) of the transcripts was carried out using NVivo 12 (QSR International Pty Ltd, Melbourne, Australia). Initial analysis was conducted by OSS. A pragmatic approach to coding was taken rather than using a theory to guide the analysis due to the researchers’ valuing the application of the study findings in the clinical setting. The transcripts were closely read line-by-line and then text segments that provided meaningful insight into the study objectives were labelled and groups of codes were subsequently added to relevant categories. Code frequencies were reviewed to identify common and novel experiences, which informed a final set of summary categories that captured the key aspects of themes identified in the transcripts given the study objectives. Following this there was a discussion between the research team to allow a diversity of inputs into reaching a consensus on key themes. Thematic saturation was determined by when no new themes appeared in the final three interviews.

### Ethics

Ethical approval for this project was granted by the University of Otago Human Research Ethics Committee (ethics reference H21/107). Consultation of participation for Māori, the indigenous people of New Zealand, was provided by the Ngai Tahu Research Consultation Committee. All participants gave informed consent to the interview, and the study was carried out in accordance with the Declaration of Helsinki.

## Results

### Participant Description

Forty-three young adults were invited to take part in an interview. Fourteen (32.5%) consented, five declined, and 24 did not respond. Most participants were women, of New Zealand European ethnicity, and used continuous or intermittently scanned glucose monitoring technology. Further demographic information of participants is presented in Table 1.

**Table 1:** Demographics of young adult participants with type 1 diabetes

Characteristics	Participants (*n* = 14)
Age (years), median [range]	*21 [18–25]*
Gender, *n* (%)	
Female	10 (71)
Male	4 (29)
Prioritized ethnicity, *n* (%)	
New Zealand European	12 (86)
Māori ^a^	2 (14)
Socioeconomic deprivation index ^b^, median [range]	3 [1–5]
Duration of diabetes (years), median [range]	14.5 [8–21]
Duration of alcohol consumption (years), median [range]	5 [2–9]
HbA1c (mmol/mol) ^c^, median [range]	60 [36–90]
HbA1c (%) ^c^, median [range]	7.6 [5.4–10.4]
Insulin regimen, *n* (%)	
CSII / MDI	12 (86) / 2 (14)
Glucose monitoring method, *n* (%)	
CBGT	6 (43)
rtCGM	5 (36)^d^
isCGM	3 (21)
Prioritised occupation, *n* (%)	
Student ^e^	10 (71)
Form of employment ^f^	4 (29)

CSII, continuous subcutaneous insulin infusion. MDI, multiple daily injections. CBGT, capillary blood glucose testing. rtCGM, real-time continuous glucose monitoring. isCGM, intermittently scanned continuous glucose monitoring. ^a^ Māori are the indigenous people of New Zealand. ^b^ Calculated by The New Zealand Deprivation Index (2018) which provides quintiles of socioeconomic status calculated using established data from home addresses with 1 representing the least deprived and 5 being the most deprived [27]. ^c^ Data was self-reported. ^d^ Three of these participants were using an advanced hybrid closed-loop insulin delivery system ^e^ Classified as an individual studying, at the time of the interview, at either a secondary or tertiary institution. ^f^ Employment refers to either part-time or full-time

### Description of Drinking Culture

Alcohol consumption within this sample reflected the normalised binge-drinking culture seen in the young adult demographic in New Zealand [18]. All participants reported that they drank socially and that they would consume above standard guidelines as per the ADA. Several participants also mentioned that they may, on occasion, drink upwards of 10 standard drinks. Some quotes related to the drinking culture observed can be found in Table 2.

**Table 2:** Quotations describing the observed drinking culture amongst the study sample

Quotations (participant no.)
*“I drink to have fun at parties, I’m not really a casual drinker. So, if I am drinking it’s for a reason which is usually to get drunk and go to a party and have fun… It’s usually like all or nothing [with drinking]” (Participant 3)*
*“Sometimes it can get messy [drinking alcohol] with the people my age, we’re all about 19–21, so we’re all still quite young and a little bit ambitious. So, some nights they’ll send it hard, and they’ll go right from the get-go, and they won’t remember anything the next morning” (Participant 5)*

### Thematic Analysis

The following four themes were identified: (i) Several sources contribute to a widely inconsistent understanding of the impact and management of alcohol consumption; (ii) Perceived inconvenience of maintaining healthy glycaemic control whilst drinking socially; (iii) Engagement in initiative-taking strategies for harm reduction occurred when convenient; and (iv) Impact of modern diabetes technology in overcoming previous burdens and promoting glycaemic safety. Representative quotes for themes and sub-themes can be found in Table 3.

**Table 3:** Summary of themes, sub-themes, and representative quotes

Themes (n)	Quotations (participant no.)
**Several sources contribute to a widely inconsistent understanding of the impact and management of alcohol consumption**	
Disparity in quality of clinical advice (9/14) Alternative sources of information (8/14) Personal experience as a learning tool (7/14) Understanding of the impact of alcohol (14/14)	*1. “My diabetes team… said to me, make sure you test, make sure you eat, make sure you have your kit on you – you pretty much never leave home without it. Stick to one thing, don’t change drinks during the middle of the night because that’s what’s gonna hit you harder” (Participant 5)* *2. “I got given… a big kind of folder-ish pamphlet thing. But they never really explained things to me, I’ve kind of had to figure it out all myself throughout these years” (Participant 6)* *3. “I have been to diabetic camp when I was a teen, and we did have a little bit of an information night about how when you first start drinking, do it in a safe environment so you know what your blood sugars are going to do before you go out to town” (Participant 10)* *4. “I guess I’m kind of used to it [learning through personal experiences] with diabetes anyway, like, trying to sort things out for myself. So, it was just, yeah, kind of just a learning curve [learning how to drink with diabetes] … I don’t mind trying to kind of figure things out, because then I know where I’m at, rather than you [medical professionals] like telling me what to do” (Participant 6)* *5. “I find that alcohol and drinking actually spikes my blood sugars – even without having sugar in it. So, it spikes my blood sugars and then I come crashing down afterwards” (Participant 9)*
**Inconvenience of maintaining healthy glycaemic control whilst drinking socially**	
Reduced frequency of monitoring (8/14) Intoxication impacting the ability to give insulin (4/14) Omittance of insulin (3/14) Removal of insulin pump (2/14)	*6. “If I did a blood test [whilst drinking], I probably wouldn’t act on it. So, there’s no point in doing it. And then it’s also the need to take and look after my blood test kit… taking it into a club or anything is just asking for it to be stolen” (Participant 3)* *7. “But usually that’s [overdosing insulin] just because of poor judgment [due to alcohol] and that sort of thing on my behalf. I look at my blood, it’s high. Okay. I take stuff [insulin] to go down, I check a bit later, it’s not gone down. And I get mad, and I just over correct [with more insulin]. And then it suddenly goes through the floor” (Participant 2)* *8. “I don’t know it’s just admin [having to inject insulin]. If I have a low when I’m out, if it’s like in town, that’s when I can pop to a Night ‘n Day [convenience store] or something. But at a party I have to ask someone for food which is just kind of embarrassing” (Participant 7)* *9. “Some nights, if I’m wearing something that doesn’t allow me to wear my pump as easily, I’ll just take it off… I’ve gone high and forgotten to put it back in” (Participant 12)*
**Engagement in initiative-taking strategies for harm reduction occurred when convenient**	
Changes to food intake (13/14) Modification of insulin dosage (9/14) Preference for low carbohydrate drinks (11/14) Peer involvement in diabetes care (14/14)	*10. “So they [other young adults] always say ‘eating is cheating’. But I always make sure that I eat beforehand have a decent meal at dinner, or before I go out anywhere [to drink]” (Participant 5)* 11. “I will make sure I have like a really carb-heavy meal [before drinking] and then not do insulin for it, just so my blood sugar is quite high and can handle whatever is gonna happen” (Participant 3) *12.“People bring, like, RTDs (ready-to-drinks) …I tend to avoid them because I know what they’re like is pretty much just lemonade and vodka, and that sort of stuff you know, that will just shoot my carbs up” (Participant 2)* *13. “I inform everyone that needs to know that I have diabetes… So, if something did go wrong, they can call an ambulance straightaway… They just know to give me sugar [If I’m low], and if I’m high they know I’ve got to inject myself with insulin” (Participant 1)*
**Impact of modern diabetes technology in overcoming previous burdens and promoting glycaemic safety**	
Increased satisfaction compared to SMBG (8/14) Additional benefits of systems (3/14)	14. “T*he sensor has been amazing… it’s just so much easier, and especially with going out and stuff. It’s just anyone can do it for me if I really needed them to. Whereas [with capillary blood glucose] testing, it’s a bit harder. It’s not available when you need it. It’s just more of a hassle, you know, having to get it out” (Participant 6)* *15. “It’s so good because I don’t need to do anything. I can just look down at my pump [paired with a CGM sensor] and I’ve got my reading. Yeah, definitely takes off a lot of like the stress of having to remember”* (Participant 12) *16. “I found with the pump [paired with a CGM sensor] it would tell me where my level was it, where it was heading, and what my levels had been like in the past” (Participant 8)* *17. “In terms of the CGM, I was doing the closed loop [automated insulin delivery system]. So, it would give me insulin if I was going high, and it would stop my basal or any insulin delivery if it was thinking I was going low… With alcohol, I guess it did help prevent those rapid crashes that sometimes I would experience. I would gradually stable out, rather than dropping really quick” (Participant 10)*

### Several sources contribute to a widely inconsistent understanding of the impact and management of alcohol consumption

A majority of the participants reported that information regarding the management of alcohol consumption was provided in some form by their diabetes clinical team. Of those who received alcohol-related information, half found that the information offered practical harm reduction benefit when applied to their drinking, including the importance of food before and after drinking and specific strategies to manage insulin dosing. However, there was some observed disparity in the quality and presentation of the data, varying from high-quality explanations to underwhelming delivery through a multitude of documents (quotes 1 and 2). One individual reported that this advice led to an increased risk of hypoglycaemia. Some of the advice provided to these young adults included ensuring food consumption before and throughout the timespan of drinking, regular glucose monitoring, and modification or omittance of insulin doses. Those who did not receive advice from their clinical team had to seek out alternative sources of education. The different sources reported included various websites, other individuals with type 1 diabetes, and family members (quote 3). The most commonly used websites for information were forms of social media with personal anecdotes; some participants did use more reputable sources such as *‘Beyond Type 1* [19]. Personal lived experience with past alcohol consumption was also rated as a very useful education tool (quote 4).

Every participant highlighted during their interview that they knew alcohol consumption was theoretically riskier for them due to their chronic condition, nonetheless, they did not let this prevent them from partaking. They all reported knowing how their blood glucose levels would react to alcohol consumption. Notably most participants found that in their experience their blood sugars would normally increase with alcohol consumption. Nearly all of these individuals then found that their blood glucose levels would begin to fall, especially overnight (quote 5).

### Inconvenience of maintaining healthy glycaemic control whilst drinking socially

Every participant reported that good glycaemic management could be a burden whilst drinking socially. This often arose due to the perceived inconvenience of typical glucose management. Twelve participants reported self-monitoring blood glucose (SMBG) by capillary blood glucose testing, of which eight reported a reduced frequency of monitoring whilst drinking. There were various reasons for this, most notably inconvenience, privacy, and redundancy (quote 6). Several of the participants also described the burden of carrying a blood test kit due to the risk of it being stolen or damaged. Reducing the frequency of monitoring was reported to increase the likelihood of hyperglycaemia. The impact of continuous glucose monitoring (CGM) devices and technology on this is discussed in a following section.

The burden of insulin management also regularly arose as another inconvenience to glycaemic management. Whilst drinking, several individuals within this study reported attempting to correct their blood glucose levels whilst being intoxicated. Four of these individuals reported over-correcting with insulin resulting in hypoglycaemia (quote 7). Some individuals would entirely avoid giving insulin due to the perceived hassle of having to manage hypoglycaemia whilst in social spaces (quote 8). Two participants also reported occasionally prioritising appearance over their management by removing their insulin pump to facilitate wearing certain outfits whilst drinking (quote 9).

The difficulty of diabetes management whilst drinking led to all the participants reporting a negative experience at some time. These negative experiences were heterogenous but oftentimes involved episodes of hypoglycaemia or hyperglycaemia as per the pattern described earlier, the experiences of losing the ability to manage their own safety, or a combination of the above. When these experiences occurred, they predominantly did not result in reported harm – defined as requiring external assistance to manage their glucose levels. However, five participants reported that their drinking has resulted in hospitalisation at some stage; two of which required an ambulance. Further, two individuals reported that they have required an intramuscular glucagon injection due to severe hypoglycaemia associated with alcohol consumption (see illustrative case below). There were no reports of other serious physical injury or diabetic ketoacidosis occurring due to alcohol consumption within this sample.

### Engagement in initiative-taking strategies for harm reduction occurred when convenient

Throughout the interviews it became apparent that all of these young adults (14/14) did make some proactive modifications to their behaviour with a perception that this would increase their safety whilst drinking.

One of the most common safety behaviours was ensuring that they ingested food throughout the period that they were drinking. A majority of the participants emphasised the importance of eating before drinking as a proactive measure to prevent hypoglycaemia (quote 10). Eating whilst drinking was less common in those interviewed but eating something after finishing drinking was seen as very important. The participants often cited that this food should be high in carbohydrates to try and counteract any effect the alcohol may have. Often in conjunction with modifying their behaviours around food, many participants reported intentionally reducing their insulin dose. Generally, these modifications were carried out to increase their glucose levels to higher than their normal target as this was perceived as being ‘safer’ and protective against hypoglycaemia (quote 11). This differs from the avoidance of insulin discussed earlier as these participants discussed these changes specifically in terms of the protective effect of such behaviours.

Another common safety behaviour was that the majority of participants reported selecting low carbohydrate alcohol beverages (quote 12), with the view to minimise harm by preventing rapidly changing glucose levels due to reduced carbohydrate ingestion.

All participants in this sample reported engaging with their peers as social supports to help manage their diabetes whilst drinking. This included making everyone aware of their condition and the basics of how to manage any potential harm (quote 13). Thereafter, most participants reported that they were confident that their peers could both identify and assist them in an emergency. This includes identifying hypoglycaemia and hyperglycaemia and knowing how to differentiate them from general intoxication. However, it is noteworthy that these peers were oftentimes also drinking with the individual.

### Impact of modern diabetes technology in overcoming previous burdens and promoting glycaemic safety

Eight of the participants interviewed had experience with modern glucose monitoring technologies such as real-time continuous glucose monitoring (rtCGM) and intermittently scanned CGM (isCGM) whilst drinking. All of them stated that they found sensors while drinking to be more convenient, as well as offering significant improvements over currently typical SMBG behaviours (quotes 14 and 15).

Notably, a majority of this group that had used modern glucose monitoring technologies found that they increased their frequency of glucose monitoring through the ease-of-use and an increased social-feasibility compared to capillary blood glucose testing. For CGM, another major benefit reported was the capacity to access trends to provide more information on blood glucose measurements, and the ability to set alarms for episodes of hypoglycaemia and hyperglycaemia (quote 16). All eight noted that these technologies made them feel safer. The illustrative case which follows (captured during the study period) depicts one of these individuals and the benefit they received from advanced diabetes technology (quote 17).

### Illustrative case (captured during study period)

The following is a participants experience that illustrates potential harms arising from alcohol consumption in a young adult with type 1 diabetes. A 23-year-old woman who manages her condition with an advanced hybrid closed loop (AHCL) insulin delivery system, spent an evening drinking socially with friends. Over a 5-hour period she reports consuming between 8 and 9 standard drinks whilst also being physically active in the form of dancing at a flat party and concert. She remained out until around 01:00 h before checking her blood glucose level (on CGM) at approximately 01:30 h and finding a measurement of around 12mmol/L. Following this she went to sleep and experienced severe hypoglycaemia and became unresponsive. Despite low glucose alarms from her AHCL system, she did not awaken and was later discovered by her parents, who were alerted by the frequent alarming, and who subsequently injected her with intramuscular glucagon to treat the hypoglycaemia.

**Fig. 1:**
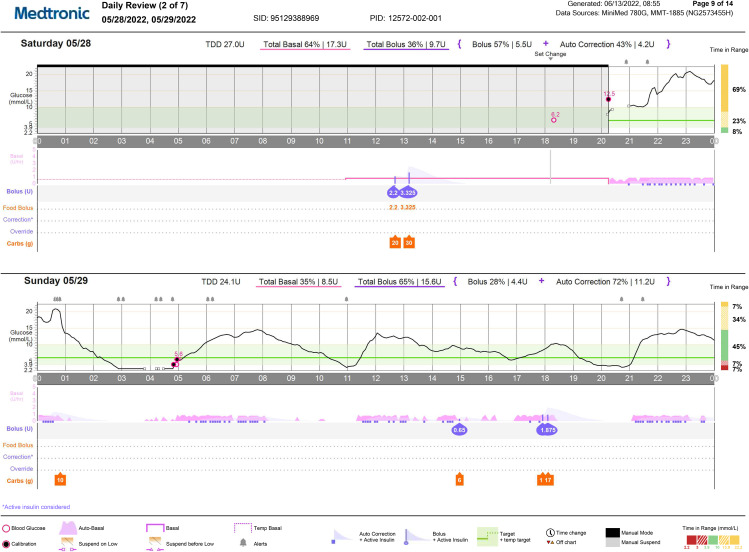
Data collected by the advanced hybrid closed-loop insulin delivery system during the evening of the event described in the illustrative case. Note: Maximal basal insulin delivery between 20:00–01:00 hours, glucose peak of 21 mmol/Ljust prior to 01:00 hours followed by a rapid drop in glucose leading to insulin delivery being suspended around 01:10 hours. Hypoglycaemia began at approximately 02:30 hours and continues until 05:00 hours upon which we see a steady increase

Figure 1 shows the CGM and insulin delivery trace from the evening when this incident occurred. As demonstrated, her glucose level continued to climb between 20:00– 01:00 h with a peak of approximately 21 mmol/L. In this period, the AHCL system was providing increased basal insulin delivery, in combination with automatic correction boluses to treat this hyperglycaemia. Just before 01:00 h, the subject entered 10 g of carbohydrates into the AHCL system that was not provided any insulin according to the systems glucose algorithm. This coincided with the period in which we can begin to see a sharp decrease in blood glucose level from 21mmol/L to 9mmol/L within one hour. In response, the AHCL system suspended insulin delivery for a 2-hour period from around 01:10 h. Low glucose alarms alerted around 03:00 and 04:00 h. Just before 05:00 h an alarm was responded to by the individual’s parents who provided an injection of intramuscular glucagon. At this stage we can begin to see the steady increase of blood glucose it reached a normal level again within 30 min.

## Discussion

This study has highlighted the potentially risky drinking behaviours of these young adults, but also how they adapt some of their behaviours to reduce the possible harms of their alcohol consumption. It has also identified the potential role of advanced diabetes technologies in reducing and managing alcohol related diabetes-harm.

Alcohol, in some countries such as New Zealand, is known to be an important factor in the social lives of young adults [20]. It is often used as a way to assist the development of social relationships; especially as this life-stage involves exploring their newfound independence [6, 21]. For those with type 1 diabetes, previous research has shown that binge-drinking is viewed as a normalised behaviour and that these young adults have a desire to participate despite the potential risks to their health [22]. This was exemplified in this study with all participants partaking in social drinking in excess of the ADA and New Zealand Ministry of Health guidelines [13, 15]. Other common risky behaviours included a reduction in the frequency of glycaemic monitoring and incoherence whilst managing insulin dosages. These behaviours often resulted in periods of hyperglycaemia followed by reactive hypoglycaemia; while a common reaction to alcohol in individuals with diabetes, the mechanisms responsible are incompletely understood but include impaired gluconeogenesis and glycogenolysis [23]. These states of reactive hypoglycaemia can be worsened by intoxication leading to a general hypoglycaemia unawareness; thus, delaying glycaemic intervention. The illustrative case presented demonstrates this risk with severe hypoglycaemia and unconsciousness as a result, despite protection available to them from automated insulin delivery low and glucose suspend. This case again emphasises the importance of pre-emptive education for managing exercise while drinking (i.e. insulin dose reduction, carbohydrate intake), preventing more extreme intoxication, and ideally close support from friends and/or family following intoxication (demonstrated by the parental intervention seen in this case). Fortunately, within this cohort no greater harms were reported, such as seizures or death, however these remain a possibility if drinking behaviour is not adequately controlled.

A review of previous studies concluded that clinical teams remain the primary educators for young adults with type 1 diabetes regarding alcohol and its potential harms [22]. Our study found that there is a disparity present in the education offered. The gulf present was highly variable; some individuals were left to fend for themselves and learn from their own harmful experiences, whilst others described receiving folders of information. Additionally, previous research using transcripts from clinic visits by adolescents and young adults with type 1 diabetes showed that only 8.5% of visits included mention of substance use, which includes alcohol [24]. These findings underline the need for a standardised level of education offered regularly to all young adults with type 1 diabetes before they start drinking alcohol, as well as for the clinical staff who would carry out such education. Furthermore, it suggests that education could involve a myriad of small feasible behaviour modifications such as what was seen by individuals in this study to reduce harm rather than focus on potentially non-feasible zero-harm interventions.

The binge-drinking culture within the young-adult age group in some places likely impacts engagement with behaviours modifications. As this study has shown, these individuals perceive themselves to be aware of the potential impact of alcohol on their glycaemic management and thus have reported making some alterations to their behaviour to reduce the harm they may face. Though it does appear that the application of these behaviour modifications is dependent on the burden such a decision would have on their overall enjoyment and engagement with drinking. As such, there were some modifications to behaviour that were widely and easily implemented despite going against social norms. One prime example of this was the generalised shirking of the social dogma of ‘eating is cheating’. Within young adulthood there is a drinking culture present in some groups, for example students in countries such as New Zealand where this sample is from, whereby the generalised aim when drinking is to become grossly inebriated. One way that is viewed to increase the likelihood of this happening is to drink alcohol on an empty stomach; hence the dogma of ‘eating is cheating’ [25, 26]. Participants in this study identified that engagement in such behaviour would likely result in hypoglycaemia, and consequently ensured that they ate a typically carbohydrate-rich meal before initiating drinking.

The data from this qualitative study also suggests that there are competing priorities at play between harm reduction and the associated burden of management behaviours. For example, harm could be reduced by consumption of fewer standards drinks, however excessive drinking can be a key component of their social life. Subsequently, these young adults would opt to drink low-carbohydrate alcohols so that excessive drinking would have a reduced impact on their glycaemia. This study also found that SMBG was burdensome in social spaces, whereas an alternative that is less invasive, such as rtCGM or isCGM, enabled appropriate monitoring behaviours. Furthermore, the illustrative case presented exhibits the other major benefits of advanced diabetes technologies such as AHCL insulin delivery systems through their capacity to alter insulin delivery in times of rapid change of glucose levels, as well as alarm the user or others to potential harm. These competing priorities need to be identified in the management plan of young adults with type 1 diabetes, and anticipatory education and management strategies need to be tailored to their specific needs. Thereafter, the potential role of advanced diabetes technologies in such contexts needs to be considered as they may offer significant benefit.

A strength of this study was the generic descriptive qualitative approach. This allowed the semi-structured interviews with the broadly diverse sample of young adults to be grounded in their unique experiences so that the research findings are more useful to diabetes care providers which subsequently influence the health and wellbeing of young adults. Additionally, interviews were carried out by a non-clinical investigator therefore discussion was minimally restricted by reluctance to discuss clinical issues. The sample of participants were diverse in age and the usage of different insulin regimens and came from different communities across three different localities: Otago, the West Coast and Greater Wellington. They also varied greatly in the types of alcohol consumed, the drinking environments they found themselves in, and in the ways that they managed their glycaemia whilst drinking. The study was also able to achieve thematic saturation as the final three interviews revealed no new themes, and so we are confident that all relevant themes were elucidated.

This study may have restricted transferability due to a high proportion of participants that were of European ethnicity, women, and a sample population with a high proportion of participants actively studying at a tertiary education facility, which is known to be associated with higher rates of a binge drinking. There was an increased rate of pump use in this sample as well due to the recruitment method of selecting individuals from those previously or currently involved in research. Also, the previous involvement in research may mean that this sample is biased towards those who are more engaged in their health. There is also a potential for re-call bias as the interviews were requesting participants to remember events that occurred through their entire time drinking, which in some cases spanned almost 10 years.

## Conclusion

This study has highlighted the experiences of young adults with type 1 diabetes who consume alcohol and the ways they engage with both the social aspects and the harms of alcohol consumption. This demographic are known to engage in binge-drinking, and for those who have type 1 diabetes they also show disproportionate rates of harm related to consumption compared to those without diabetes. However, this study has exhibited that these harms may be worsened by a disparity in anticipatory education surrounding alcohol, and thus suggests that there is a need for further development in this area to reduce future harm. This study also indicates that education may need to be individually tailored to consider the competing interests of sociability and harm-reduction to find more feasible interventions and support strategies to be implemented. Diabetes technology may have an important complementary role in these strategies to promote safe glucose control during alcohol consumption.

### Electronic supplementary material

Below is the link to the electronic supplementary material.

Supplementary Material 1

## Data Availability

The transcripts analysed in this study are not publicly available due to privacy concerns for the participants but may be made available from the corresponding author on reasonable request.
