# Supplementary material for: Knowledge, safety, and impact of alcohol consumption in young adults with type 1 diabetes mellitus: a qualitative study
Source: BMC Endocr Disord. 2023 Oct 20;23:229. doi: 10.1186/s12902-023-01471-7 (PMC10588012; doi:10.1186/s12902-023-01471-7)
Supplement: Supplementary file 1 — Supplementary Material 1 [file 12902_2023_1471_MOESM1_ESM.docx]

**Knowledge, safety, and impact of alcohol consumption in young adults with type 1 diabetes: a qualitative study**

**Interview Guide**

Basic introduction, then start with open question such as “Tell me about the role of alcohol in your current life?”

Survey/interview guide (topics to be covered):

Knowledge of risks of alcohol

Where does knowledge come from, what sources

Awareness of prior education / outline of prior education

Drinking behaviour (how often/how many standard drinks/…)

Kinds of drinks? (alcohol/sugar levels?)

Glucose monitoring behaviour during/after drinking?

Discuss Negative (positive) experiences / health experiences with drinking and diabetes

What diabetes related preparations are done before/during drinking? (extra bolus/ carbs intake/eating behaviour)

What changes to usual care are done after drinking? Directly after/ the following day?

Awareness of their peers for diabetes? Role of their family/parents/caregivers in safety

Individuals in social group that are trained in assistance? 🡪 How to spot difference between drunk vs low?
